# Prisoners' understanding and experiences of parole

**DOI:** 10.1002/cbm.2178

**Published:** 2020-11-17

**Authors:** Lynn Kelly, Gill McIvor, Karen Richard

**Affiliations:** ^1^ School of Education and Social Work University of Dundee Dundee UK; ^2^ School of Applied Social Science University of Stirling Stirling UK; ^3^ Parole Board for Scotland Edinburgh UK

**Keywords:** early release, parole, participation, prisoners, Scotland

## Abstract

**Background:**

In Scotland, as elsewhere, there has been growing political and public interest in the function and process of parole accompanied by a lack of empirical research on the operation and effectiveness of parole.

**Aims:**

Against the backdrop of a Scottish Government review of parole aimed, among other things, at improving the transparency of the process, the aim of the study was to explore the experiences of prisoners seeking early release on licence.

**Methods:**

In conjunction with the Scottish Prison Service a national survey was conducted of 197 long‐term prisoners who had experience of seeking early release on parole.

**Findings:**

The survey revealed that prisoners did not have a clear understanding of parole and often did not feel fully engaged in the process.

**Conclusions:**

It is argued that better support for prisoners prior to, during and following parole hearings might foster their increased engagement and alleviate some of the anxiety associated with the parole process.

## 1 BACKGROUND

It is more than 50 years since the introduction of discretionary early release on parole in Scotland in 1968. Since then, prison populations have raised internationally reflecting a growth in the numbers of short‐ and long‐term prison sentences. In Scotland short term prisoners (with sentences of up to 4 years) are automatically released after serving half of their sentence. Decisions regarding the discretionary release of long‐term (those serving sentences of 4 years and over), and indeterminate sentence prisoners, and the recall to prison of those who are deemed to have breached the conditions of their early release are the responsibility of the Parole Board for Scotland (PBS) which consists of legal and general members appointed by the Cabinet Secretary for Justice.

In Scotland, the parole process varies according to the category of prisoner. When a prisoner becomes eligible for consideration for parole (at the half way point in the case of determinate sentence prisoners or at the end of the ‘punishment part’ or tariff in the case of those given indeterminate sentences) a dossier of information and reports is compiled by the prison and submitted to the prisoner and Parole Scotland (the organisation responsible for managing the work of the PBS). Prisoners serving indeterminate sentences and those serving extended sentences who have been recalled to prison and are in the ‘extension’ part of their sentence have their cases heard at a tribunal—usually face to face but in some cases via videoconferencing—comprising a legal chair and two other PBS members. Hearings for determinate sentence prisoners take the form of paper‐based casework meetings also involving a legal chair and two other PBS members who may reach a decision based on existing information, defer consideration for further information or, if it is considered necessary in the interests of fairness and justice, recommend that an oral hearing of the case be convened to which relevant witnesses may be cited to give evidence.

Prisoners attend—usually with legal representation—and are encouraged to participate actively in tribunals and oral hearings by answering questions from Board members but they are not legally required to do so. Evidence from the prisoner is preceded by an overview of progress by the Lifer Liaison Officer (LLO) or Early Release Liaison Officer (ERLO) and by questioning of any witnesses—such as social workers or psychologists—who have been cited to attend. If the decision of the Board is to not recommend for direct release, it will indicate when the next review of the case should take place: this should be no longer than the statutory review period (which is 2 years in the case of indeterminate sentence prisoners). The parole process in Scotland is, it has been suggested, characterised by ‘complex interrelationships that exist between different elements of the system’ (McAra, 2000, p. 130).

Given the diversity of the characteristics of prisoners seeking parole, the inherent complexity of the decision‐making process and the far‐reaching consequences for those seeking parole, their families and the victims of crime (Padfield, 2019) it is vital that parole policy and practice is, as far as possible, empirically informed. However, the processes associated with parole, the experiences of those involved and the outcomes of parole decision‐making have been largely neglected in contemporary criminological literature (Griffin, 2018). Recent media, public and political interest generated by controversies such as the Worboys case in England and Wales—in which a Parole Board decision to release a convicted rapist resulted in political intervention to overturn the decision and dismiss the Chair of the Board—has brought into relief the lack of research on parole (Carr & Blay, 2019). It is unclear why this important topic has largely evaded academic scrutiny. Naylor and Schmidt (2010) have suggested that the lack of transparency of Parole Boards is likely to have been a contributory factor and may explain the 1970 finding (Bilton & Bottomley, 2012) that prisoners seeking release perceived the process to be overly secretive. Arguably recent heightened interest has generated a push toward greater transparency and engagement by all stakeholders in the parole process. In Scotland this has included the publication of information about parole processes and outcomes and guidance for victims and professionals. Consideration is also being given to the publication of decision minutes and further involvement of victims in the parole process. It is in this context that the present study was commissioned.

Existing parole research has tended to focus on parole decision‐making (e.g. Hutton & Levy, 2002; McAra, 1998), the post‐release experiences of prisoners (e.g. Armstrong & Durnescu, 2016; Dünkel et al., 2018) and processes of recall (e.g. Barry, 2019; Weaver, Tata, Munro, & Barry, 2012; Padfield, 2012). In contrast to other areas of penological inquiry, where the experiences of those who have been sentenced are seen as critical to our understanding, there has been relatively little research into serving prisoners' experiences of parole. Existing studies tend to be somewhat dated, relatively small scale or focused on experiences of imprisonment more broadly. They have, however, identified aspects of imprisonment that may impinge upon the wellbeing and mental health of prisoners seeking early release.

Griffin and Healy's (2019) study of life sentence prisoners in Ireland has identified the ‘pains' of the indeterminate sentence and the uncertainty that it brings for prisoners along with ‘palpable exasperation at the delays in the parole process and the perception that the system lacked transparency’ (p. 136). In a very small‐scale study the prisoners interviewed by Gadhia and Aslan (2018) reported feeling anxious prior to the hearing but subsequently found the parole process to be generally positive and believed that they had been listened to. In contrast, Martin (2018)—a life sentence prisoner—has argued that ‘the whole of the Parole Board process is focused on satisfying a set of criteria on that day’ and that the main thing to be gained from it was a sense that ‘once you've been through it you can face anything’ (p. 48).

In an early study by Bilton and Bottomley in 1970, prisoners expressed a sense of confusion about what was expected of them and in relation to the predictability of Parole Board decision making, reportedly finding the system ‘unjust and, more particularly, “too secret”’ (Bilton & Bottomley, 2012, p. 17). This latter study appears to have been prompted by Lord Hunt, the then chairman of the Parole Board for England and Wales, who was concerned that ‘the average prisoner feels that he does not have enough “say,” and is therefore apathetic about parole’ (p. 15). There has clearly been a long standing interest by Parole Boards regarding the engagement or otherwise of those seeking parole but little research has addressed this issue.

### 1.1 Aims of the study

Given the importance of parole for the parties concerned, it is crucial to understand the experiences of the many actors in the process, including prisoners who are eligible to participate in the parole process. This is consistent with calls for greater accountability of public services and enhanced public participation in decision‐making (Public Services Commission, 2011). The concept of participation is not, however, unproblematic. The findings of the study by Jackson, Kelly, and Leslie (2020) raise important questions about the extent to which participants can be considered to meaningfully participate in formal legal processes. In the context of parole, tensions exist between the judicial function of the Parole Board and the informed rights‐based discourses of prisoner participation. As a consequence of discursive practices and bureaucratic arrangements, the voice of the professional is always likely to dominate and be imbued with a higher value (Buckley, Carr, & Whelan, 2011; Jackson et al., 2020) resulting in the exercising of ‘soft power’ that creates uncertainty and affects the thinking and behaviour of prisoners (Crewe, 2011).

The Scottish Government (2018) has recently embarked upon a review of parole in Scotland. Amidst public scrutiny and heightened political and policy interest in parole, it is important that any changes that are introduced are informed by relevant research (Jones, 2018) and that the ‘human’ face of parole should be subject to greater attention (Padfield, 2019). To begin to address these concerns and to gain an understanding of prisoners’ experiences as a means of informing future policy and practice, the Parole Board for Scotland (PBS) authorised a survey, undertaken in conjunction with the Scottish Prison Service (SPS), of prisoners who had experience of the parole process.

The survey focused on prisoners serving long term determinate and indeterminate sentences. The questionnaire (a copy of which can be obtained from the lead author) explored their understanding and experiences of the parole process with a view to identifying areas for potential improvement. It was developed by members of the PBS research group (three of whom are the authors of this article) in consultation with colleagues in the research and parole units at SPS headquarters and with two prisoners from the Open Estate who were completing higher education qualifications. While not speaking directly to issues of participation, the survey was designed to explore prisoners’ views about their level of engagement and participation in the process. Despite the acknowledged limitations of the methodology, the survey provides a useful starting point upon which other studies can build.

## 2 METHODOLOGY

The questionnaire consisted of a mixture of fixed choice questions and Likert scales. There were also opportunities for prisoners to provide additional written comments, though few did so. Prisoners eligible to be included in the survey were identified from the SPS database on 1 March 2019. The questionnaire was distributed to prisoners who were identified as having participated in the parole process during the previous 2 years. About 520 questionnaires were distributed by SPS and 197 at least partially completed questionnaires were returned, representing a respectable response rate of 38%. The survey sought prisoners’ views about preparation for their hearing, their understanding of the contents of the dossier and their significance, and their experiences of the parole hearing itself. Analysis was undertaken using SPSS to produce descriptive statistics, with resulting percentages based upon the numbers of prisoners responding to each question (rather than the total sample since it was not usually possible to determine what a nil response signified). Some analysis was also undertaken to identify whether the experiences of different categories of prisoners varied. To preserve their anonymity prisoners were asked to provide a limited amount of demographic information which would not enable their identification.

Questionnaires were sent directly to prisoners with a covering letter explaining the purpose of the research and the steps taken to preserve confidentiality. A sealable envelope in which responses could be returned to the research team at SPS headquarters via internal mail was also included. The majority of respondents (97%) were male and the sample was evenly divided among those under and over 45 years of age. While 14% identified as first offenders, 42% indicated that they had 11 or more previous convictions. The largest number of responses was from life sentence prisoners (45%) and prisoners subject to an Order for Lifelong Restriction (OLR) (similar to the indeterminate sentence for public protection (IPP) in England and Wales) (35%). The remainder consisted of extended sentence prisoners (13%), prisoners serving long‐term determinate sentences (5%) and those serving other unspecified sentences (2%). Only 12 of the respondents indicated that they had previously been granted parole, with 8 of this group stating that they had been recalled on at least one occasion.

It is important to acknowledge the limitations of the methodology. For example, the fact that the questionnaire was distributed to serving prisoners meant that the target audience were, by inference, prisoners whose parole applications had not been successful (or who had been successful on a previous occasion but subsequently recalled). Moreover, the sample did not include prisoners who had been transferred under mental health legislation using a Transfer for Treatment Direction (TTD) to an NHS facility. The views expressed cannot, therefore, be assumed to be representative of all prisoners with experience of the parole process. In addition, although efforts were made to keep the language used clear and understandable, levels of literacy among prisoners are typically low (Creese, 2015). This is likely to have precluded the participation of many prisoners along with the small percentage of prisoners whose first language is not English. Practical and resource considerations meant that the questionnaires were distributed by prisons and not by the researchers themselves. It was not therefore possible to ascertain whether prisoners who might have required support to read and complete the questionnaire received it. This might also have contributed to the limited information prisoners gave to elaborate upon their fixed choice responses.

## 3 FINDINGS

### 3.1 Preparing for the hearing

Life sentence prisoners (74%) were more likely than those subject to OLRs (52%) or extended sentences (46%) to indicate that they understood the parole process (*X* ^2^, 2 *df* = 9.71, *p* < 0.01). Less than one half of the respondents (44%) indicated that they had been able to access information about the parole process before their case was considered by the Board. Prisoners’ knowledge about parole was most commonly obtained from legal representatives (45%), fellow prisoners (39%) and SPS staff (34%). While only 10% of prisoners indicated that they had been provided with a leaflet about the parole process, 39% suggested that they would have found a leaflet useful. Although other prisoners often provided information about parole, relatively few prisoners (13%) considered such a source to be useful: it is possible that prisoners resorted to advice from other prisoners in the absence of other authoritative sources of information.

Probably because they had experienced them in person, the majority of prisoners indicated that they understood what oral hearings (74%) and tribunals (78%) were. Extended sentence prisoners were less likely than life sentence and OLR prisoners to understand what these types of hearings involved, presumably because they were less likely to have experienced them. By contrast, less than one‐third (32%) of the sample understood what casework hearings—which involve solely a paper‐based consideration of the case—were. Given the centrality of the legal tests for parole to the decision whether or not to release a prisoner, it was concerning to find that only 39% of prisoners apparently understood the legal tests that the Parole Board is required to apply.

### 3.2 The dossier

The parole dossier is a substantial document consisting, in most cases, of the indictment, trial judge report, antecedents, reports by a range of different professionals (including post‐programme reports for prisoners who have completed prison‐based intervention programmes) and the minutes of previous hearings. While most of the sample indicated that they had received a copy of their dossier in time to prepare for their hearing, almost one in five (18%) reported that they did not. Of some concern, given the volume of sensitive confidential material contained in parole dossiers, was the finding that only 63% of prisoners indicated that they were able to keep their dossier in a safe place prior to their tribunal. Since prisoners were not required to indicate in which prison they were located it is not clear whether this was an issue across the estate or specific to certain institutions.

Legal, medical and psychological language is over‐represented in parole documentation, meaning that reading and comprehension of the dossier presents many challenges. It was perhaps surprising, therefore, that the majority of prisoners (86%) indicated that they had been able to read their dossier and that the information was presented in a way that they could understand. This may be because survey participants had better than average literacy skills and that difficulties in reading and understanding their dossier may be more widespread among prisoners as a whole. While most of the 32 prisoners who could not read their dossier indicated that they had received help to do so from other sources (mostly prison staff, legal representatives and other prisoners) some (6) stated that they had received no assistance in this respect.

Few prisoners reported that they had felt involved in the preparation of their dossier (35%). One mechanism through which prisoners can make an active contribution to the dossier is through the provision of self‐representations. Most of the sample (70%) asserted that they had not received any guidance on why they might want to submit self‐representations and only 58% of those who responded indicated that they had done so. Among this group, 27% had received no assistance in preparing their self‐representations.

When considering the contents of the dossier, while the majority of prisoners felt that the Response in Custody Reports completed by a prison officer (55%), health reports (55%) and previous Parole Board minutes (52%) accurately represented their progress, only 40% believed this to be the case for social work reports while 44% felt that the Early Release or Lifer Liaison Officer report was accurate. Although the majority of prisoners (66%) indicated that they did not have an opportunity to address inaccuracies in the dossier prior to or during the parole hearing, it is not clear in how many of these cases inaccuracies had actually existed.

Prisoners were asked to indicate how important they believed the various contents of the dossier to be in informing the decision of the Parole Board. Respondents were most likely to consider risk assessments and the index offence (53%), post‐programme reports (46%), disciplinary reports and drug testing history (39%) as being of very high importance. Health reports, prison intelligence and prisoner self‐representations (30%) were least likely to be considered very important in the context of Parole Board decision‐making. Most (21) of the prisoners whose dossiers had contained victims, representations and who expressed a view (31) believed that these representations affected the decision‐making of the Board.

### 3.3 Experience of parole hearings

The majority of prisoners had experience of attending a parole hearing in person, with only 13% indicating that they had not done so (presumably because their case had been considered instead at a casework hearing). The majority of hearings had been conducted face‐to‐face although in 20% of cases the most recent hearing had been conducted by videolink. Anxiety was the emotion most commonly experienced prior to a hearing (50% of respondents) followed by frustration (35%) and hope (24%). Some of those seeking release (11%) said that they ‘felt nothing’ before the hearing took place.

While only 48% of those seeking release considered that they were given adequate information to identify a suitable legal representative in advance of their hearing, 63% believed that they had adequate legal representation to prepare for the hearing and 62% believed that they were adequately represented during the hearing itself. However, that means that more than one‐third of the sample did not consider their legal representation to have been adequate. For example, fewer than half of the sample (44%) believed that their case for release had been adequately presented to the Board. This may be because in Scotland there is a relatively limited pool of legal practitioners who specialise in and are experienced in parole work and who are therefore better placed to advise their clients and present effective submissions to the Board.

During the hearing itself 64% of respondents felt that they properly understood what was going on and 81% indicated that they understood the questions put by the Board. Life sentence prisoners (70%), who tended to have more experience of participating in tribunals, were more likely than those sentenced to an OLR (61%) or extended sentences (29%) to say they understood what was happening during their hearing (*X* ^2^, 2 *df* = 10.11, *p* < 0.01). While around one half of the sample (51%) believed that they had had an adequate opportunity to address the Board on matters important to them, 75% were of the view that support in addition to a legal representative would have been helpful.

As previously indicated, respondents understood risk to be a key factor in Parole Board decision‐making. The majority of respondents (68%) were of the view that undertaking offence‐focused programme work had helped them to understand their offending and therefore to reduce their risk. However only 37% felt that they had been given an opportunity at their hearing to demonstrate that their risk had reduced since coming into custody, while 35% were of the view that the Parole Board had accurately determined the risk that they posed, possibly reflecting differing understandings of the nature and significance of risk among prisoners and professionals. This, along with not having subsequently received any help in understanding the Parole Board's decision (31%) may help to explain why the most common emotion experienced after a hearing was frustration (55%). By contrast, in the aftermath of hearings 27% reported feeling anxious and only 19% were hopeful of a positive outcome.

## 4 DISCUSSION

Given the importance of the parole process, the survey highlighted significant gaps in respondents’ understanding of parole. The limited understanding about many aspects of the process has the potential to undermine the ability of those seeking release to effectively prepare for and present their case to the Board. Given the increasing emphasis on transparency with respect to the parole process, the provision of clear written information to prisoners sufficiently in advance of their hearing would enable them to better understand what will be required of them and what they need to take into account to address Board members’ likely concerns. Parole Scotland has made recent efforts to enhance the transparency of the parole process to professionals and to the public—including the victims of crime—and would be well placed to extend this to prisoners through the production of written material for prisoners, that is, informative and easy to understand.

Many prisoners will require assistance to read and understand their dossiers in preparation for their Parole hearing. The ways in which legal documents might be rendered unreadable through the use of ‘legalese’ by those outwith the profession has been documented (Wogalter, 1999) while others have argued that professionals fail to differentiate between audiences in their written communications with little regard for the literacy capabilities of the intended recipient (Green, Duncan, Barnes, & Oberklaid, 2003). The low levels of literacy among prisoners makes it likely that for many much of the content of dossiers will not be easily understood (Prison Reform Trust, 2013). It is argued that further attention needs to be paid to ensuring that the contents of dossiers are as clear and accessible as possible to prisoners with varying levels of literacy. If prisoners are unable to understand the content of their dossier their preparations and representations are likely to be seriously compromised.

Victim representations in dossiers were often perceived by prisoners as having a direct bearing on decision‐making; however, their role in this process remains unclear. It has been argued that there has been a legal and political ‘rediscovery’ of the victim in recent times (Braun, 2019a). Of particular relevance here is the United Nations Declaration of Basic Principles of Justice for Victims of Crime and Abuse of Power, adopted in 1985, which requires Member States to consider the position of victims in criminal justice systems and how they might be awarded more rights to participate in legal proceedings (United Nations, 1985). The release of those convicted of serious crimes undoubtedly has an impact on those who have been affected by them. However, as we have previously argued, notions of participation are contested and not without problem (Jackson et al., 2020). Perhaps for this reason a review of international responses to victim participation in the context of early release concluded that victim participation remains limited (Braun, 2019b) and there is still no consensus as to how victim rights can be upheld in the context of early release proceedings.

Further consideration needs to be given to how prisoners’ effective participation in the parole process can be increased. This is particularly important as a means of promoting perceptions of procedural justice (Tyler, 1990)—prisoners’ perceptions that they have been listened to and treated fairly by justice practitioners—which in turn may enhance the perceived legitimacy of Parole Board decisions and prisoners’ willingness to comply with the licence conditions imposed upon them to manage their behaviour when they are released. Bottoms (2001) has argued that compliance and effectiveness of community supervision are inextricably linked while Robinson and McNeill (2008) suggest that longer term desistance is more likely to be associated with substantive (as opposed to simply formal) compliance which is characterised by active engagement and co‐operation of those under supervision and dependent on the perceived legitimacy of the supervisory requirements with which they are expected to comply.

Direct participation in parole hearings may allow prisoners to feel a sense of ownership of the process, by providing an opportunity to put their case across, but for many it will also be a stressful experience. It is unclear how this experience affects prisoners’ behaviour in the immediate aftermath or how much support is available to them during this period. The finding that relatively few prisoners had been assisted to understand the Parole Board's decision suggests that limited support is routinely available to them at this time.

Having a clearer understanding of the parole process may alleviate some of the anxiety that prisoners experience prior to, during and following the hearing, although for many prisoners this process will still cause significant anxiety. This is of relevance given the relatively high incidence of prisoners who experience difficulties with their mental health during their sentence (Bowler, Phillips, & Rees, 2018; Haney, 2003) and following their release on parole (Fedock et al., 2018). There is a risk that mental health problems may be exacerbated by the process and associated? uncertainty of parole.

Experience from the mental health field points to the potential benefits of enhanced support to prisoners at various stages in the parole process through advocacy and mentoring. For example, under the Mental Health (Care and Treatment) (Scotland) Act 2003, anyone with a mental disorder has the right to access an independent advocate. An independent advocate is able to give support and help to enable a person to express their own views about their care and treatment. Within the Mental Health Tribunal for Scotland (2018) (MHTS), a patient can nominate a Named Person (NP) who can look after their interests under the terms of mental health legislation. They have status under the Act and are entitled to legal representation (Scottish Government, 2019).

Patients in the mental health system may also be represented by a curator‐ad‐litem if they are not fit to instruct. This has particular significance for prisoners with dementia where they do not have capacity. It is understood that proposals are currently being considered to amend the Parole Board (Scotland) Rules 2001 to enable the appointment of a curator‐ad‐litem under these circumstances. More generally, mentors or advocates could have a key role to play not just in supporting prisoners during their hearings, but also in helping them to better understand the different stages of the parole process and supporting them throughout. While support of this kind may not eradicate the uncertainty and anxiety associated with the process of seeking parole, it may at least reduce some of the impact in this regard. However, given the highly sensitive nature of material contained in dossiers, consideration needs to be given to issues of confidentiality and the potential for disclosure or misuse of information in deciding who should have access, under what circumstances and to what ends. For similar reasons, further consideration needs to be given to ensuring that prisoners are able to store their dossiers without them potentially being accessible to other prisoners and staff.

As we have already argued, professionals need to recognise the imbalances of power that exist in processes such as parole that can serve as barriers to the effective participation of those seeking release. A more democratic and participatory process is not only in the interests of those seeking parole but is also in the interests of those who have been affected by serious crime. The present survey was a small but important step toward involving prisoners and addressing some of the inherent imbalances in the system. We are, as previously discussed, aware of the methodological limitations of our approach. The survey has been useful in highlighting prisoners’ understanding and experiences of the parole process and identifying potential areas for improvement, but surveys do not permit deeper and more nuanced insights and they arguably place limits on participation by predetermining the questions that are posed. For this reason, interviews or focus groups with prisoners would be a useful mechanism for exploring in more depth some of the key issues highlighted by the present survey results.

## CONFLICT OF INTERESTS

The authors have no conflict of interest to declare.

## DATA AVAILABILITY STATEMENT

The data that support the findings of this study are available on request from the corresponding author. The data are not publicly available due to privacy or ethical restrictions.
